# Supplementary material for: Transcriptomic Analysis of Skin Tissue Reveals Molecular Mechanisms of Thermal Adaptation in Cold-Exposed Lambs
Source: Animals (Basel). 2025 May 13;15(10):1405. doi: 10.3390/ani15101405 (PMC12108302; doi:10.3390/ani15101405)
Supplement: Supplementary file 1 [file animals-15-01405-s001.zip › animals-3606668-supplementary.pdf]

Table S1. Differences in various characteristics of Hulunbuir and Hu lambs at different temperatures.

Characteristic	15°C	–20°C	Hu	Hulunbuir	SEM	*p*-value		
						Temperature	Breed	Interaction
WGL (cm)	1.1 ± 0.04	1.7 ± 0.10	1.3 ± 0.09	1.4 ± 0.15	0.09	< 0.001	0.243	0.046
CWMFD (μm)	53.9 ± 2.11	51.4 ± 2.24	55.0 ± 2.53	50.3 ± 1.46	1.52	0.411	0.130	0.374
FWMFD (μm)	20.5 ± 0.58	21.1 ± 0.42	21.7 ± 0.40	19.8 ± 0.41	0.35	0.344	0.004	0.262
FW (%)	54.4 ± 3.05	50.8 ± 2.42	55.8 ± 2.23	49.4 ± 2.93	1.94	0.356	0.107	0.631
WY (g)	0.3 ± 0.02	0.3 ± 0.03	0.3 ± 0.02	0.3 ± 0.03	0.02	0.140	0.037	0.139
WGC (%)	10.0 ± 0.67	10.6 ± 0.35	9.6 ± 0.50	11.0 ± 0.49	0.38	0.366	0.061	0.183
HFD (/mm^2)	38.7 ± 2.39	33.0 ± 1.45	33.5 ± 1.74	38.1 ± 2.31	1.51	0.044	0.094	0.208
ET (μm)	15.2 ± 0.65	18.7 ± 0.79	16.5 ± 0.60	17.3 ± 1.15	0.64	0.002	0.431	0.050
ADG (g/d)	105.5 ± 17.03	59.6 ± 10.44	75.2 ± 17.44	89.8 ± 14.14	11.05	0.037	0.480	0.286
BT (mm)	5.4 ± 0.36	5.3 ± 0.38	5.3 ± 0.25	5.3 ± 0.46	0.26	0.766	0.968	0.143
HCW (kg)	20.6 ± 0.38	19.7 ± 0.41	20.3 ± 0.39	20.0 ± 0.45	0.29	0.105	0.562	0.277
DP (%)	51.3 ± 0.60	52.1 ± 0.66	51.9 ± 0.61	51.5 ± 0.67	0.44	0.410	0.637	0.091
RT (°C)	38.8 ± 0.04	38.1 ± 0.09	38.4 ± 0.14	38.4 ± 0.12	0.09	< 0.001	0.923	0.923
RR (breaths/min)	22.4 ± 1.42	16.0 ± 0.84	18.7 ± 1.36	19.7 ± 1.75	1.09	0.001	0.526	0.213

WGL: wool growth length; CWMFD: coarse wool mean fibre diameter; FWMFD: fine wool mean fibre diameter; FW: the proportion of fine wool fibres; WY: wool yield; WGC: wool grease content; HFD: hair follicle density; ET: epidermis thickness; ADG: average daily gain; BT: backfat thickness; HCW: hot carcass weight; DP: dressing percentage; RT: rectal temperature; and RR: respiratory rate. *p*-values derived from either two-way ANOVA model. The interaction refers to the combined effect of temperature and breed on the variable.

Table S2. Differences in various characteristics of the Hulunbuir and Hu lambs at different temperatures.

Characteristic	–20°C		15°C		*p*-value			
	**HU–20**	**HB–20**	**HU+15**	**HB+15**	**HU+15 vs. HU–20**	**HB+15 vs. HB–20**	**HU–20 vs HB–20**	**HU+15 vs HB+15**
WGL (cm)	1.5 ± 0.12	1.8 ± 0.13	1.1 ± 0.08	1.0 ± 0.46	0.028	<0.001	0.096	0.310
CWMFD (μm)	52.4 ± 3.84	50.4 ± 2.71	57.6 ± 3.29	50.2 ± 1.52	0.333	0.948	0.677	0.074
FWMFD (μm)	21.7 ± 0.69	20.4 ± 0.35	21.8 ± 0.48	19.2 ± 0.67	0.903	0.147	0.144	0.014
FW (%)	55.0 ± 2.59	46.7 ± 3.32	56.7 ± 3.90	52.1 ± 4.89	0.721	0.385	0.085	0.484
WY (g)	0.3 ± 0.15	0.4 ± 0.03	0.3 ± 0.03	0.3 ± 0.03	0.999	0.074	0.025	0.630
WGC (%)	10.4 ± 0.55	10.9 ± 0.49	8.8 ± 0.71	11.2 ± 0.91	0.115	0.767	0.577	0.078
HFD (/mm^2)	32.4 ± 2.20	33.6 ± 2.11	34.7 ± 2.87	42.7 ± 3.06	0.549	0.041	0.702	0.092
ET (μm)	17.3 ± 0.64	20.0 ± 1.20	15.8 ± 0.97	14.6 ± 0.87	0.240	0.006	0.076	0.370
ADG (g/d)	41.2 ± 7.16	77.9 ± 16.44	109.3 ± 27.16	101.6 ± 23.66	0.042	0.435	0.074	0.837
BT (mm)	4.8 ± 0.27	5.7 ± 0.70	5.8 ± 0.32	5.0 ± 0.64	0.052	0.518	0.308	0.308
HCW (kg)	19.5 ± 0.47	19.8 ± 0.72	21.1 ± 0.39	20.2 ± 0.62	0.030	0.732	0.736	0.221
DP (%)	53.0 ± 0.73	51.1 ± 0.98	50.8 ± 0.70	51.9 ± 0.99	0.054	0.574	0.147	0.379
RT (°C)	38.1 ± 0.16	38.1 ± 0.10	38.8 ±0.07	38.8 ± 0.04	0.004	<0.001	0.918	1.000
RR (breaths/min)	16.5 ± 1.37	15.5±1.08	20.8±2.05	24.0 ±1.89	0.122	0.004	0.559	0.284

WGL: wool growth length; CWMFD: coarse wool mean fibre diameter; FWMFD: fine wool mean fibre diameter; FW: the proportion of fine wool fibres; WY: wool yield; WGC: wool grease content; HFD: hair follicle density; ET: epidermis thickness; ADG: average daily gain; BT: backfat thickness; HCW: hot carcass weight; DP: dressing percentage; RT: rectal temperature; and RR: respiratory rate. *p*-values derived from LSD post hoc tests.

Table S3. Summary of sequencing data quality.

Sample	M Total seqs[a]	% Mapped[b]	M Reads mapped[c]	% GC[d]	Error rate[e]
HU–20-1[f]	46.7	93.9	43.9	50	0.83
HU–20-2	46.8	94.2	44.1	50	0.75
HU–20-3	45.4	93.7	42.6	51	0.93
HU–20-4	45.5	94.2	42.9	50	0.84
HU–20-5	48.0	94.3	45.3	50	0.84
HB–20-1	47.0	94.6	44.5	51	0.58
HB–20-2	48.2	94.3	45.5	50	0.72
HB–20-3	47.2	94.4	44.5	50	0.79
HB–20-4	46.7	94.9	44.3	50	0.73
HB–20-5	48.2	94.5	45.6	50	0.78
HU+15-1	47.9	94.8	45.4	49	0.75
HU+15-2	46.7	94.1	44.0	50	0.81
HU+15-3	45.5	93.9	42.8	49	0.71
HU+15-4	47.2	94.3	44.5	50	0.81
HU+15-5	45.8	94.8	43.4	49	0.76
HB+15-1	45.2	94.5	42.7	50	0.79
HB+15-2	45.6	94.4	43.1	50	0.81
HB+15-3	47.6	94.3	44.9	50	0.80
HB+15-4	45.5	95.2	43.3	49	0.59
HB+15-5	47.8	94.1	44.9	50	0.91

[a]The total number of sequences in the BAM file (millions).

[b]The percentage of mapped reads.

[c]The number of mapped reads in the BAM file (millions).

[d]The average GC% of the transcripts.

[e]The mismatch rate (NM) / bases mapped (CIGAR).

[f]HU+15: Hu lambs at 15℃; HU–20: Hu lambs at -20℃; HB+15: Hulunbuir lambs at 15℃; and HB–20: Hulunbuir lambs at –20℃.

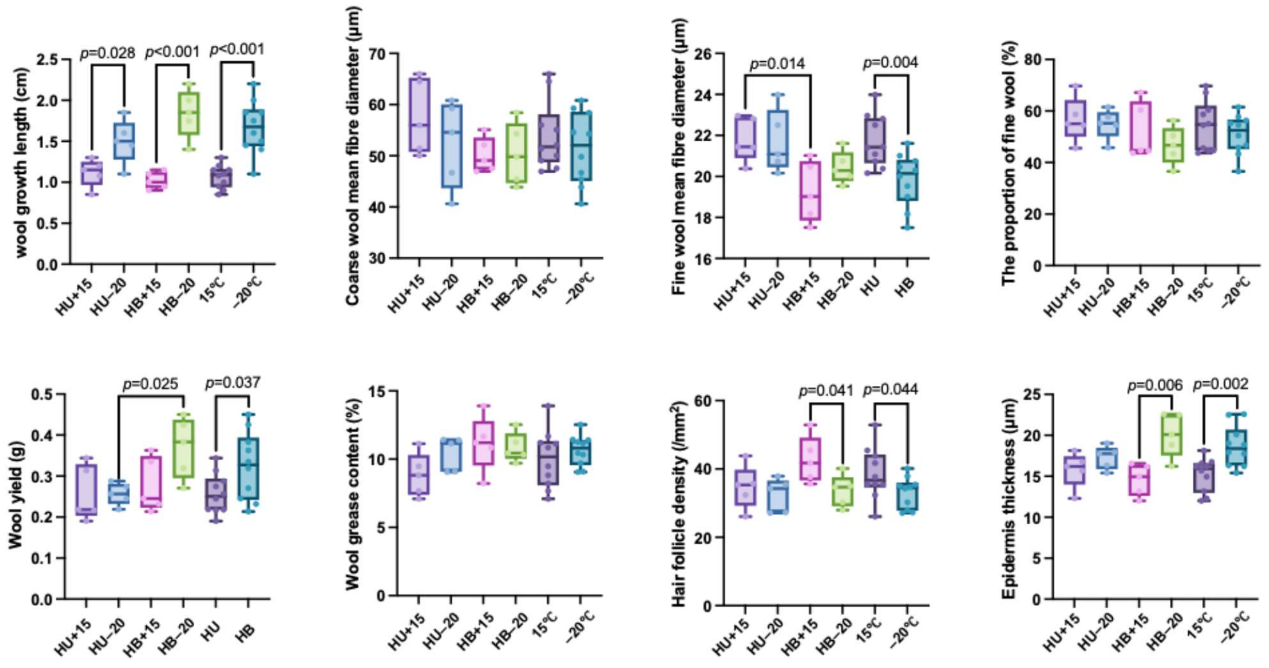

Figure S1. Wool and skin phenotypes in Hulunbuir lambs and Hu lambs at different temperatures. Hulunbuir at –20°C (HB–20), Hulunbuir at 15°C (HB+15), Hu at –20°C (HU–20), and Hu at 15°C (HU+15). The *p*-values are derived from either two-way ANOVA models or LSD post hoc tests.

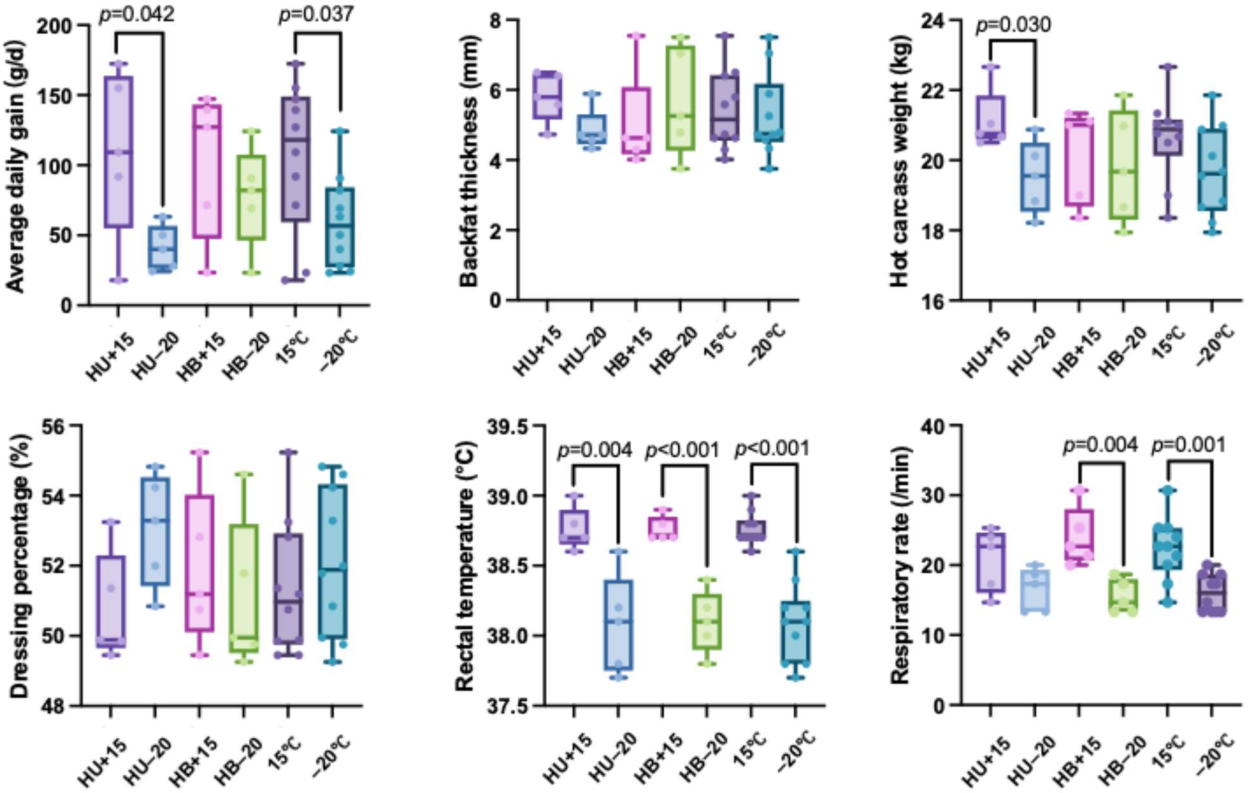

Figure S2. Selected physiological traits in Hulunbuir and Hu lambs at different temperatures. Hulunbuir at –20°C (HB–20), Hulunbuir at 15°C (HB+15), Hu at –20°C (HU–20), and Hu at 15°C (HU+15). The *p*-values are derived from either two-way ANOVA models or LSD post hoc tests.

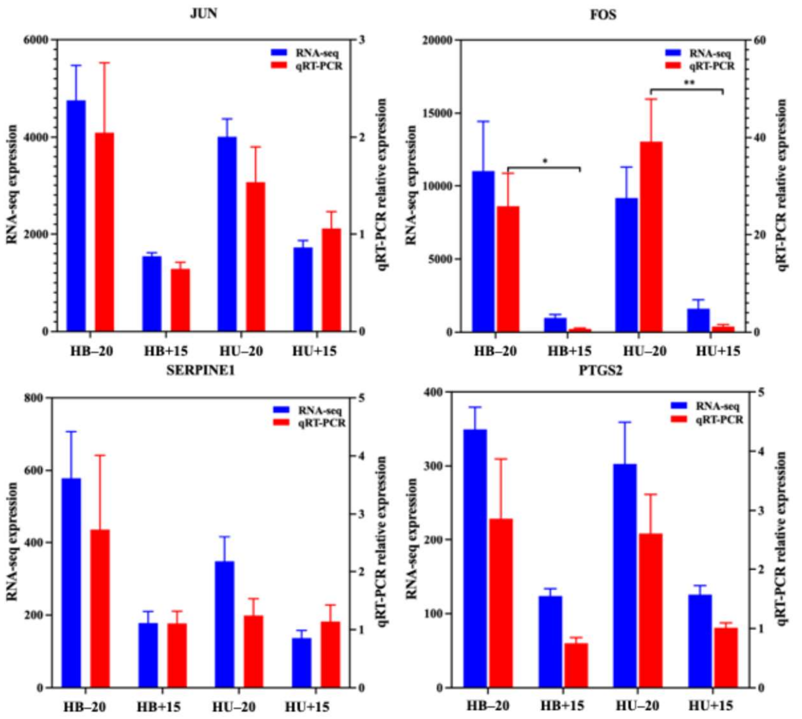

Figure S3. Comparisons of the RT-qPCR and RNA-Seq analyses in the four groups. Hulunbuir at –20°C (HB–20), Hulunbuir at 15°C (HB+15), Hu at –20°C (HU–20), and Hu at 15°C (HU+15). * indicates $p < 0.05$, ** indicates $p < 0.01$.
